# Bio-inspired Analytical Heuristics to Study Pine Wilt Disease Model

**DOI:** 10.1038/s41598-020-60088-1

**Published:** 2020-02-26

**Authors:** Muhammad Ozair, Takasar Hussain, Aziz Ullah Awan, Adnan Aslam, Riaz Ahmad Khan, Farhad Ali, Fatima Tasneem

**Affiliations:** 1 Department of Mathematics, COMSATS University Islamabad, Attock Campus, Attock, Pakistan; 2 Department of Mathematics, University of the Punjab, Lahore, Pakistan; 3 School of Electrical Engineering and Computer Science (SEECS), National University of Sciences and Technology (NUST), Islamabad, Pakistan; 4 School of Mechanical and Manufacturing Engineering (SMME), National University of Sciences and Technology (NUST), Islamabad, Pakistan; 5 Institute of Numerical Sciences, Kohat University of Science and Technology (KUST), Kohat, KPK Pakistan

**Keywords:** Plant sciences, Mathematics and computing

## Abstract

This paper portrays the dynamics of pine wilt disease. The specific formula for reproduction number is accomplished. Global behavior is completely demonstrated on the basis of the basic reproduction number ${{\boldsymbol{R}}}_{{\boldsymbol{o}}}$. The disease-free equilibrium is globally asymptotically stable for ${{\boldsymbol{R}}}_{{\boldsymbol{o}}}{\boldsymbol{ < }}{\bf{1}}$ and in such a case, the endemic equilibrium does not exist. If ${{\boldsymbol{R}}}_{{\boldsymbol{o}}}$ exceeds one, the disease persists and the unique endemic equilibrium is globally asymptotically stable. Global stability of disease-free equilibrium is proved using a Lyapunov function. A graph-theoretic approach is applied to show the global stability of the unique endemic equilibrium. Sensitivity analysis has been established and control strategies have been designed on the basis of sensitivity analysis.

## Introduction

Forests play a vital role in human life, so it is essential to set up safety measures for the protection of infected trees. The trees do not just give greenery to nature, but also supply a charming climate for humans. Pine wilt disease (PWD) rapidly spreads in the tree, and within a few weeks, the tree starts exhibiting the symptoms and moves towards death quickly. Now, this disease has become a threat to the forest ecosystem and to pine forests. It is a very dangerous and wild disease among pine trees with huge economic losses which disturb the development of social sustainability. There is no way to cure the pine tree once infected. Consequently, prevention is the standard technique^1^.

The early occurrence of PWD goes back to $1905$. During the $1930s$ the annual loss of pines increased from $30,\,000\ {m}^{3}$ to $200,\,000\ {m}^{3}$, and during 1940s destructions were estimated at volumes of $400,\,000\ {m}^{3}$ every year. The struggle to control the disease was abandoned during World War II, which resulted in an expeditious increase of infected pine trees and an immense loss of $1,\,230,\,000\ {m}^{3}$ in 1948. Considerable efforts lessened the annual loss to $400,\,000\ {m}^{3}$ during the 1950s. Elimination of dead trees through tumbling and blazing was used as the basic control method at that time. In the $1970s$, annual losses exceeded $1$ million ${m}^{3}$ again. Prevalent growth in the infested region was identified as one of the most characteristic features of the epidemic at that time. Considerable timber loss, $2.4$ million ${m}^{3}$, was recorded in $1979$^2^.

*Bursaphelenchus xylophilus* is the nematode that is the main cause of this disease. It was first described in 1971 as the causal agent of PWD of native pines in Japan^3^. *Monochamus alternatus* (pine sawyer beetle) is the vector for this parasite. The initial notable symptom of PWD is the reduction in the emission of oleoresin from wounds of bark. A further indication is the conversion of needle color. It becomes light grayish green to yellowish-green then yellowish-brown. Lastly, fully brown when tree capitulates to the malady^4,5^.

Three types of transmission of PWD are discovered:

- First, when infected adult beetles fly to susceptible pine trees and start maturation feeding, transmit nematode in it, this is known as the primary transmission^6^.
- Secondary transmission develops when mature females lay eggs on dead or dying, newly cut pine trees^7^.
- The third is the horizontal transmission of the nematode. It happens through the mating of male and female bark beetles^8^.

Numerous mathematical models have been designed to analyze the transmission dynamics of PWD. In 1999, Yoshimura *et al*.^9^ studied that there is a minimum density of pine under which the invasion of the disease reduces and a minimum density of pine increases unjustifiably with increment in the termination rate. Takasu *et al*.^10^ analyzed the reliance on parasite rate of range expansion on the extermination rate of the beetle, primary pine density, and the vector dispersal capacity. Mechanistic interactions are considered in^11^, and it is demonstrated that the Allee effect can appear in the beetle dynamics due to the need for beetles to contact pine trees at least twice to imitate auspiciously. Mathematical models based on ordinary differential equations have been analyzed in^12–16^. Shi and Song^17^ studied the mathematical model for the transmission of pine wilt disease by considering two routes. Firstly, healthy pine trees are infected through the maturation feeding of infectious bark beetles and secondly, when mature females lay eggs on infected dead or dying trees. However, they did not consider the third transmission of nematodes among bark beetles through mating that has been suggested by Togashi and Arakawa^18^.

In this paper, we revisit the model given in^17^ and included the following features:

- Transmission occurs in the vector population through the mating.
- The optimal control problem has been designed by performing a sensitivity analysis of basic reproduction number ${R}_{o}$, infected host ${I}^{\ast }$ and infected beetles ${Y}^{\ast }.$

The aim of this paper is to study new features of the models:

1. To show the global behavior of equilibria.
2. Identification of sensitive parameters for reproduction number and endemic levels of infectious classes.
3. Design the optimal control strategy on the basis of sensitivity analysis.

## Model Framework with Flow Diagram

We formulate the mathematical model comprises of susceptible host pine trees $S(t)$, infected host pine trees $I(t)$, susceptible vector beetles $X(t)$ and infected beetles $Y(t)$. The usual mode of nematode transmission between pine trees and bark beetles develops as a result of maturation feeding. The pine sawyers have pinewood nematodes when they emerge from infected pines. However, the vectors may also be directly infected during mating. The population of host pine trees at time ${\bf{t}}$ is represented by ${\mathbb{W}}$, and whole vector adult beetles are indicated by ${\mathbb{A}}$. Thus, ${\mathbb{W}}=S+I$ and ${\mathbb{A}}=X+Y.$

Let $a$ be the constant recruitment rate of *Monochamus alternatus*, $b$ be the increase rate of pines, $\mu$ be the fatality rate of *Monochamus alternatus*. Pines are infected by the *Monochamus alternatus* having *Bursaphelenchus xylophilus Nickle*. This contact process is assumed to follow the bilinear incident rate $\beta YS$. Bark beetles, which do not carry nematode is infected by *Bursaphelenchus xylophilus Nickle* through infectious pines and this contact process is represented by the incidence rate $kIX$. *Monochamus alternatus* is infected through mating and the contact process is denoted by ${\beta }_{1}XY.$ Let $\alpha$ be the felling rate of susceptible pines, $\delta$ be the exploitation rate of pines which have *Bursaphelenchus xylophilus*. Here we assume that $\delta  > \alpha$.

With these assumptions, the model can be demonstrated by the following system of non-linear differential equations:

1 $$\begin{array}{lll}\frac{dS}{dt} & = & b-\beta YS-\alpha S,\\ \frac{dI}{dt} & = & \beta YS-\delta I,\\ \frac{dX}{dt} & = & a-kIX-{\beta }_{1}XY-\mu X,\\ \frac{dY}{dt} & = & kIX+{\beta }_{1}XY-\mu Y.\end{array}$$

**Figure 1:**
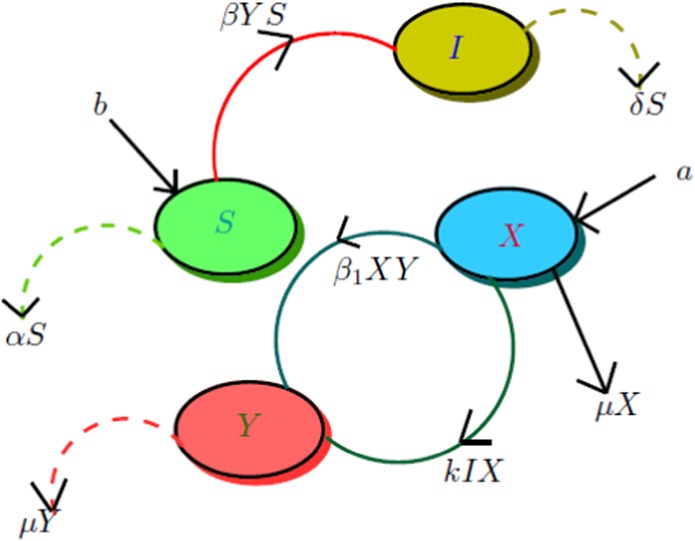
Flow chart of the model (1).

The flow diagram of the model (1) is shown in Fig. 1.

As the model deals with beetle and tree populations, it is clear that all the state variables will be non-negative for non-negative initial conditions, that is, ${\bf{t}}\ \ge \ 0$. The total population of both the species satisfies the equations:

2 $$\begin{array}{lll}\frac{d{\mathbb{W}}}{d{\bf{t}}} & = & b-\alpha S-\delta I,\\ \frac{d{\mathbb{A}}}{d{\bf{t}}} & = & a-\mu X-\mu Y.\end{array}$$

Clearly, $\xi$ = $\{(S,I,X,Y)\in {R}_{+}^{4}| \frac{b}{\delta }\ \le \ S+I\ \le \ \frac{b}{\alpha },0\ \le \ X+Y\ \le \ \frac{a}{\mu }\}$ is positively invariant, the global attractor is contained in $\xi$ and the model (1) is dissipative.

## Equilibria and Basic Reproduction Number

Direct calculations show that the model (1) always attains a disease-free equilibrium (DFE) given by ${E}_{o}=\left(\frac{b}{\alpha },0,\frac{a}{\mu },0\right)$. The dynamics of the malady is characterized by the basic reproduction number that is defined to be the average number of secondary infections, which are accomplished by an infected individual in a totally susceptible population. This enables us to find whether an infectious disease will prevail through population or not. We use the next-generation method to calculate the basic reproduction number.

Let $x={({x}_{1},{x}_{2},...,{x}_{n})}^{T}\in {R}^{n}$ and $y={({y}_{1},{y}_{2},...,{y}_{m})}^{T}\in {R}^{m}$ be the population in disease and non-disease compartments, respectively, $\widehat{F}={({\widehat{F}}_{1},{\widehat{F}}_{2},...,{\widehat{F}}_{n})}^{T}$ and $\widehat{V}={({\widehat{V}}_{1},{\widehat{V}}_{2},...,{\widehat{V}}_{n})}^{T},$ where ${\widehat{F}}_{i}$ shows the rate of new infections in *ith* disease class and ${\widehat{V}}_{i}$ shows the transition terms, for example recovery and death in the *ith* disease class^19^. ${R}_{\circ }$ is acquired by choosing the dominant eigenvalue (spectral radius) of $\widehat{F}{\widehat{V}}^{-1}$, where $\widehat{F}=\left[\frac{\partial ({\widehat{F}}_{i}({E}_{\circ }))}{\partial ({{\bf{x}}}_{j})}\right]$ and $\widehat{V}=\left[\frac{\partial ({\widehat{V}}_{i}({E}_{\circ }))}{\partial ({{\bf{x}}}_{j})}\right].$ The infected compartments are $I$ and $Y.$ Therefore,

$$\left[\begin{array}{l}{\widehat{F}}_{1}\\ {\widehat{F}}_{2}\end{array}\right]=\left[\begin{array}{l}\beta YS\\ kIX+{\beta }_{1}XY\end{array}\right],\ \left[\begin{array}{l}\widehat{{V}_{1}}\\ \widehat{{V}_{2}}\end{array}\right]=\left[\begin{array}{l}\delta I\\ \mu Y\end{array}\right].$$

Thus,

$$\widehat{F}=\left(\begin{array}{ll}0 & \beta S\\ kX & {\beta }_{1}X\end{array}\right),\ \widehat{V}=\left(\begin{array}{ll}\delta  & 0\\ 0 & \mu \end{array}\right),$$

which gives

$$\widehat{F}{\widehat{V}}^{-1}=\left[\begin{array}{ll}0 & \frac{\beta b}{\alpha \mu }\\ \frac{ak}{\delta \mu } & \frac{{\beta }_{1}a}{{\mu }^{2}}\end{array}\right].$$

Since ${R}_{\circ }$ is the spectral radius of $\widehat{F}{\widehat{V}}^{-1}$, therefore,

$${R}_{o}=\frac{\frac{{\beta }_{1}a}{{\mu }^{2}}+\sqrt{{\left(\frac{{\beta }_{1}a}{{\mu }^{2}}\right)}^{2}+4\frac{\beta kab}{\alpha \delta {\mu }^{2}}}}{2}.$$

For ${R}_{\circ } > 1$, the system (1) has a unique endemic equilibrium (EE) ${E}^{* }$ = $({S}^{* },{I}^{* },{X}^{* },{Y}^{* })$, where

3 $$\begin{array}{lll}{S}^{* } & = & \frac{b}{\beta {Y}^{* }+\alpha },\\ {I}^{* } & = & \frac{\beta {Y}^{* }}{\delta }\frac{b}{\beta {Y}^{* }+\alpha },\\ {X}^{* } & = & \frac{a\delta (\beta {Y}^{* }+\alpha )}{kb\beta {Y}^{* }+\delta (\beta {Y}^{* }+\alpha )({\beta }_{1}{Y}^{* }+\mu )},\end{array}$$

and ${Y}^{* }$ is uniquely calculated from the equation

4 $$A{Y}^{{* }^{2}}+B{Y}^{* }+C=0,$$

where

$$\begin{array}{lll}A & = & {\beta }_{1}\mu \delta \beta ,\\ B & = & \mu bk\beta +{\mu }^{2}\delta \beta +{\beta }_{1}\mu \delta \alpha -{\beta }_{1},\\ C & = & {\mu }^{2}\delta \alpha \left[1-\left(\frac{{\beta }_{1}a}{{\mu }^{2}}+\frac{abk\beta }{{\mu }^{2}\delta \alpha }\right)\right].\end{array}$$

Noting that $A > 0$ while the sign of $C$ coincides with that of ${R}_{\circ }$ so that $C < 0$ if and only if ${R}_{\circ } > 1$. Thus we conclude that if ${R}_{\circ } > 1$, the model (1) has unique EE given as ${E}^{* }=({S}^{* },{I}^{* },{X}^{* },{Y}^{* })$.

## Stability of Equilibria

We investigate the global behavior of the system (1) around DFE. For this, we construct the Lyapunov function.

### Theorem 1.

For ${R}_{\circ }\ \le \ 1$, the DFE of the system (1) is globally asymptotically stable in  $\xi$.

*Proof*. Consider the Lyapunov function

$$L=\frac{1}{\delta }I+\frac{\mu }{ka}Y.$$

Taking time derivative of $L$, we have

$$\begin{array}{lll}{L}^{{\prime} } & = & \frac{1}{\delta }{I}^{{\prime} }+\frac{\mu }{ka}{Y}^{{\prime} },\\  & = & \frac{1}{\delta }\left(\beta Y\frac{b}{\alpha }-\delta I\right)+\frac{\mu }{ka}\left(kI\frac{a}{\mu }+{\beta }_{1}\frac{a}{\mu }Y-\mu Y\right),\\  & = & \left(\frac{1}{\delta }\frac{\beta b}{\alpha }+\frac{\mu }{ka}{\beta }_{1}\frac{a}{\mu }-\frac{\mu }{ka}\mu \right)Y+\left(\frac{\mu }{ka}k\frac{a}{\mu }-\frac{1}{\delta }\delta \right)I,\\  & \le  & \left(\frac{1}{\delta }\frac{\beta b}{\alpha }+\frac{{\beta }_{1}}{k}-\frac{{\mu }^{2}}{ka}\right)Y,\\  & = & \frac{{\mu }^{2}}{ka}\left(\frac{ka\frac{\beta b}{\alpha \delta }+ka\frac{{\beta }_{1}}{k}}{{\mu }^{2}}-1\right)Y,\\  & = & \frac{{\mu }^{2}}{ka}\left(\frac{\beta bka}{{\mu }^{2}\alpha \delta }+\frac{a{\beta }_{1}}{{\mu }^{2}}-1\right)Y.\end{array}$$

${L}^{{\prime} }\ \le \ 0$ if and only if ${R}_{\circ }\ \le \ 1$. Furthermore, ${L}^{{\prime} }=0$ if and only if $I=Y=0$. Thus, it is easy to check that the largest compact invariant set for the system (1) is singleton ${E}_{\circ }$. By Lasalle’s Invariance Principle^21^, ${E}_{\circ }$ is globally asymptotically stable (GAS) in $\xi$.□

We now will show that the unique EE is GAS using the graph-theoretic approach. For further terminologies, the readers are referred to^20^.

### Theorem 2.

*The unique positive disease-present equilibrium point has global asymptotic stability in the interior of* $\xi$.*Proof*. Let

$$\begin{array}{lll}{{\mathbb{D}}}_{1} & = & S-{S}^{* }-{S}^{* }{\rm{ln}}\,\frac{S}{{S}^{* }},\\ {{\mathbb{D}}}_{2} & = & I-{I}^{* }-{I}^{* }{\rm{ln}}\,\frac{I}{{I}^{* }},\\ {{\mathbb{D}}}_{3} & = & X-{X}^{* }-{X}^{* }{\rm{ln}}\,\frac{X}{{X}^{* }},\\ {{\mathbb{D}}}_{4} & = & Y-{Y}^{* }-{Y}^{* }{\rm{ln}}\,\frac{Y}{{Y}^{* }}.\end{array}$$

Differentiating and using the inequality $1-x+{\rm{ln}}\,x\ \le \ 0$ for $x < 0$, we have

$$\begin{array}{lll}{{\mathbb{D}}}_{1}^{{\prime} } & = & \left(1-\frac{{S}^{* }}{S}\right){S}^{{\prime} }\\  & = & \left(1-\frac{{S}^{* }}{S}\right)\left(b-\beta YS-\alpha S\right)\\  & = & \left(1-\frac{{S}^{* }}{S}\right)\left(\beta {Y}^{* }{S}^{* }+\alpha {S}^{* }-\beta YS-\alpha S\right)\\  & = & \left(1-\frac{{S}^{* }}{S}\right)\left(\beta {Y}^{* }{S}^{* }-\beta YS\right)+\left(1-\frac{{S}^{* }}{S}\right)\left(\alpha {S}^{* }-\alpha S\right)\\  & = & \beta {Y}^{* }{S}^{* }\left(1-\frac{{S}^{* }}{S}\right)\left(1-\frac{YS}{{Y}^{* }{S}^{* }}\right)+\alpha {S}^{* }\left(1-\frac{{S}^{* }}{S}\right)\left(1-\frac{S}{{S}^{* }}\right)\\  & = & \beta {Y}^{* }{S}^{* }\left(1-\frac{YS}{{Y}^{* }{S}^{* }}-\frac{{S}^{* }}{S}+\frac{Y}{{Y}^{* }}\right)+\alpha {S}^{* }\left(\frac{S-{S}^{* }}{S}\right)\left(\frac{{S}^{* }-S}{{S}^{* }}\right)\\  & = & \beta {Y}^{* }{S}^{* }\left(1-\frac{{S}^{* }}{S}-\frac{YS}{{Y}^{* }{S}^{* }}+\frac{Y}{{Y}^{* }}\right)-\alpha {S}^{* }\frac{{\left(S-{S}^{* }\right)}^{2}}{S{S}^{* }}\\  & \le  & \beta {Y}^{* }{S}^{* }\left(-{\rm{ln}}\,\frac{{S}^{* }}{S}-\frac{YS}{{Y}^{* }{S}^{* }}+\frac{Y}{{Y}^{* }}\right)-\alpha {S}^{* }\frac{{\left(S-{S}^{* }\right)}^{2}}{S{S}^{* }}\\  & \le  & \beta {Y}^{* }{S}^{* }\left({\rm{ln}}\,\frac{S}{{S}^{* }}-\frac{YS}{{Y}^{* }{S}^{* }}+\frac{Y}{{Y}^{* }}\right),\\  & \le  & \beta {Y}^{* }{S}^{* }\left({\rm{ln}}\,\frac{S}{{S}^{* }}+{\rm{ln}}\,\frac{Y}{{Y}^{* }}-{\rm{ln}}\,\frac{Y}{{Y}^{* }}-\frac{YS}{{Y}^{* }{S}^{* }}+\frac{Y}{{Y}^{* }}\right)\\  & \le  & \beta {Y}^{* }{S}^{* }\left({\rm{ln}}\,\frac{SY}{{S}^{* }{Y}^{* }}-{\rm{ln}}\,\frac{Y}{{Y}^{* }}-\frac{YS}{{Y}^{* }{S}^{* }}+\frac{Y}{{Y}^{* }}\right)\\  & := & {a}_{14}{{\bf{G}}}_{14}.\end{array}$$

Similarly,

$$\begin{array}{lll}{{\mathbb{D}}}_{2}^{{\prime} } & \le  & \beta {Y}^{* }{S}^{* }\left({\rm{ln}}\,\frac{{I}^{* }}{I}\frac{YS}{{Y}^{* }{S}^{* }}-{\rm{ln}}\,\frac{YS}{{Y}^{* }{S}^{* }}+\frac{YS}{{Y}^{* }{S}^{* }}-\frac{{I}^{* }}{I}\frac{YS}{{Y}^{* }{S}^{* }}\right)\\  & := & {a}_{23}{{\bf{G}}}_{23},\\ {{\mathbb{D}}}_{3}^{{\prime} } & \le  & k{I}^{* }{X}^{* }\left({\rm{ln}}\,\frac{X}{{X}^{* }}\frac{I}{{I}^{* }}-\frac{IX}{{I}^{* }{X}^{* }}-{\rm{ln}}\,\frac{I}{{I}^{* }}+\frac{I}{{I}^{* }}\right)+{\beta }_{1}{X}^{* }{Y}^{* }\\  &  & \left({\rm{ln}}\,\frac{X}{{X}^{* }}\frac{Y}{{Y}^{* }}-\frac{XY}{{X}^{* }{Y}^{* }}-{\rm{ln}}\,\frac{Y}{{Y}^{* }}+\frac{Y}{{Y}^{* }}\right)\\  & := & {a}_{31}{{\bf{G}}}_{31}+{a}_{34}{G}_{34},\\ {{\mathbb{D}}}_{4}^{{\prime} } & \le  & k{I}^{* }{X}^{* }\left({\rm{ln}}\,\frac{IX{Y}^{* }}{{I}^{* }{X}^{* }Y}-\frac{IX{Y}^{* }}{{I}^{* }{X}^{* }Y}+\frac{IX}{{I}^{* }{X}^{* }}-{\rm{ln}}\,\frac{IX}{{I}^{* }{X}^{* }}\right)\\  &  & +{\beta }_{1}{X}^{* }{Y}^{* }\left(\frac{XY}{{X}^{* }{Y}^{* }}-{\rm{ln}}\,\frac{XY}{{X}^{* }{Y}^{* }}+{\rm{ln}}\,\frac{X}{{X}^{* }}-\frac{X}{{X}^{* }}\right)\\  & := & {a}_{43}{G}_{43}+{a}_{42}{G}_{42}.\end{array}$$

**Figure 2:**
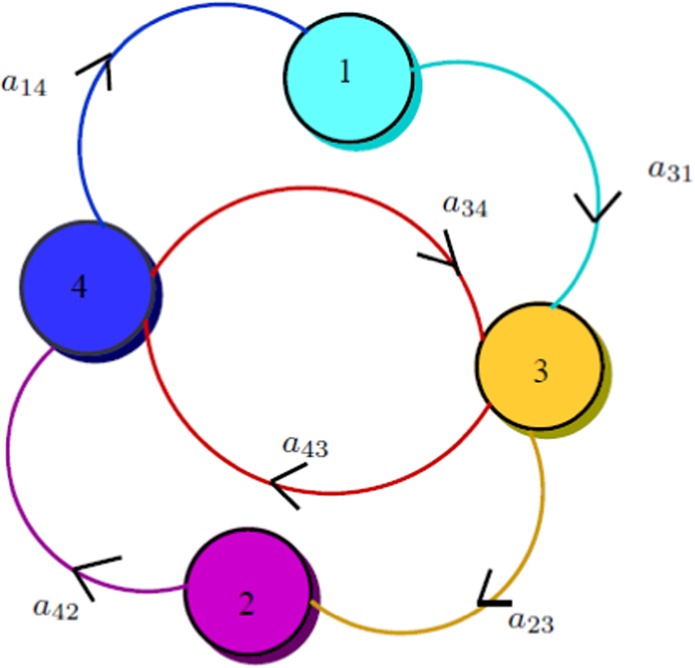
Weighted diagraph.

A weighted digraph is constructed with four vertices and six arcs, which is shown in Fig. 2:

It is easy to see that there are three cycles and along each cycle $${{\bf{G}}}_{31}+{{\bf{G}}}_{43}+{{\bf{G}}}_{14}=0$$, $${{\bf{G}}}_{31}+{{\bf{G}}}_{23}+{{\bf{G}}}_{42}+{{\bf{G}}}_{14}=0,$$$${{\bf{G}}}_{23}+{{\bf{G}}}_{42}+{{\bf{G}}}_{34}=0$$. Thus, by Theorem (3.5) from^20^, there exist $${c}_{1}$$, $${c}_{2}$$, $${c}_{3}$$, $${c}_{4}$$, such that $${\mathbb{D}}={c}_{1}{{\mathbb{D}}}_{1}+{c}_{2}{{\mathbb{D}}}_{2}+{c}_{3}{{\mathbb{D}}}_{3}$$. $$+{c}_{4}{{\mathbb{D}}}_{4}$$From Theorem (3.3) given in^20^, it follows that

$$\begin{array}{lll}{c}_{3}{a}_{31} & = & {c}_{1}{a}_{14},\\ {c}_{4}{a}_{42} & = & {c}_{2}{a}_{23}.\end{array}$$

Thus,

$${\mathbb{D}}={D}_{1}+{D}_{2}+\frac{\beta {Y}^{* }{S}^{* }}{k{I}^{* }{X}^{* }}{D}_{3}+\frac{\beta {S}^{* }}{{\beta }_{1}{X}^{* }}{D}_{4},$$

is the Lyapunov function of the system (1). Using this Lyapunov function and LaSalle’s invariance principle^21^, it follows that ${E}^{* }$ is GAS in int ($\xi$).□

## Sensitivity Analysis

The main thing for an infectious disease is to study its capability to enter in a population. To check that which factors are responsible for the expanse and the existence of the disease, we shall carry out the sensitivity analysis. In this analysis, we explain the following features:

- How the endemic level of host and vector population is affected by changing the values of parameters?
- The measurement of the corresponding change in the state variable when a parameter changes.

### Effect of parameters on the endemic level of infectious hosts

**Figure 3:**
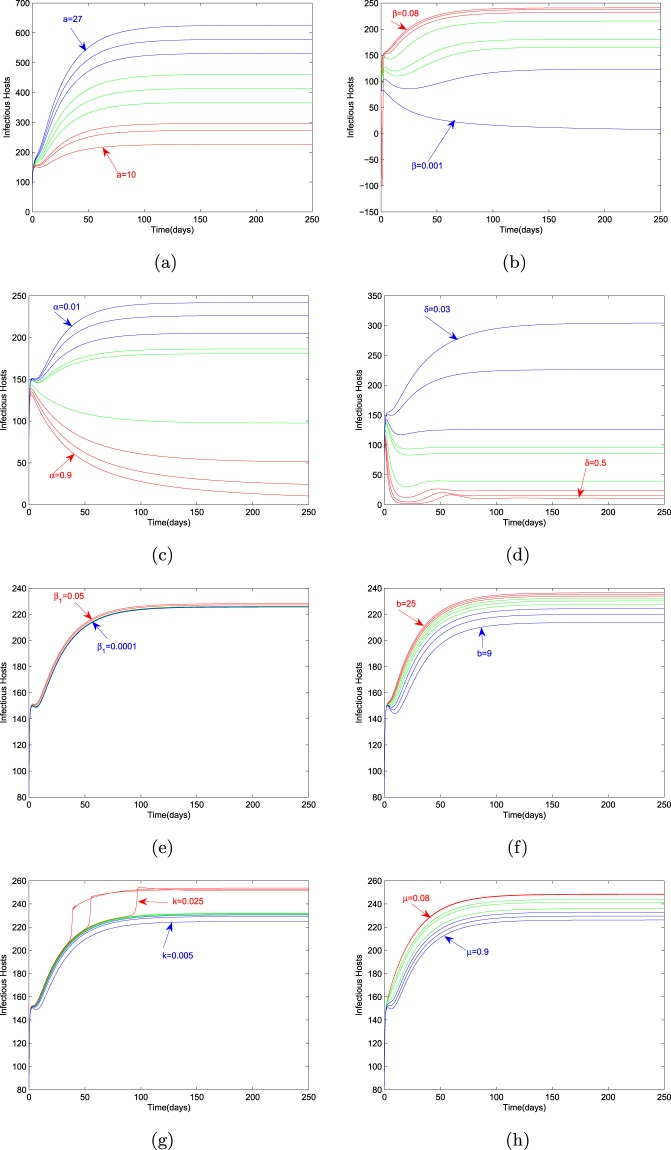
Variation in the endemic level of *I*^∗^ with respect to model parameters.

 Figure 3 shows the endemic level of infectious hosts for different values of the parameters. Graphs explore that parameters $a,\beta ,{\beta }_{1},b,k$ are directly related to ${I}^{* }$ and inversely related to $\alpha ,\delta ,\mu$. It can also be observed that considerable change occurs in the value of ${I}^{* }$ by an increment or reduction of $a,\beta ,\alpha$ and $\delta$. However, when we calculate the percentage difference of the values of ${I}^{* }$ corresponding to the percentage difference of the parameter values, the most sensitive parameter is $\beta$, which is the transmission rate of the *Bursaphelenchus xylophilus Nickle* through the maturation feeding of infectious *Monochamus alternatus*. The value of this percentage difference of the endemic level is $416195$. It means that we should focus on the reduction of the transmission rate of nematode while designing the control policy. Percent increment or reduction in the value of ${I}^{* }$ corresponding to all parameters is given in Table 1.

**Table 1:** Sensitivity of ${I}^{* }$ for all model parameters.

Parameters	Initial	Final	Difference	Percentage	Initial	Final	Difference	Percentage	${{\bf{C}}}_{{\bf{5}}}{\boldsymbol{/}}{\bf{91.11111111}}$	${{\bf{C}}}_{{\bf{9}}}{\boldsymbol{* }}{{\bf{C}}}_{{\bf{10}}}$
		Value		Difference	Value of ${I}^{* }$	Value of ${I}^{* }$		Difference		
$a$	10	27	17	170	220	620	400	181.8181818	1.865853659	339.2461197
$\beta$	0.001	0.08	0.079	7900	5	245	240	4800	86.70731707	416195.122
$\alpha$	0.9	0.01	$-0.89$	$-98.88888889$	10	240	230	2300	$-1.085365854$	$-$2496.341463
$\delta$	0.5	0.03	-0.47	$-$94	7	304	297	4242.857143	$-$1.031707317	$-$4377.38676
${\beta }_{1}$	0.0001	00.05	0.0499	49900	206	210	4	1.941747573	547.6829268 k8	1063.461994
$b$	9	25	16	177.7777778	214	236	22	10.28037383	1.95122	20.05927
$k$	0.005	0.025	0.02	400	224	256	32	14.28571429	4.390243902	62.71777003
$\mu$	0.9	0.08	$-$0.82	-91.11111111	224	246	22	9.821428571	$-$1	$-$9.821428571

### Effect of parameters on the endemic level of infectious bark beetles

**Figure 4:**
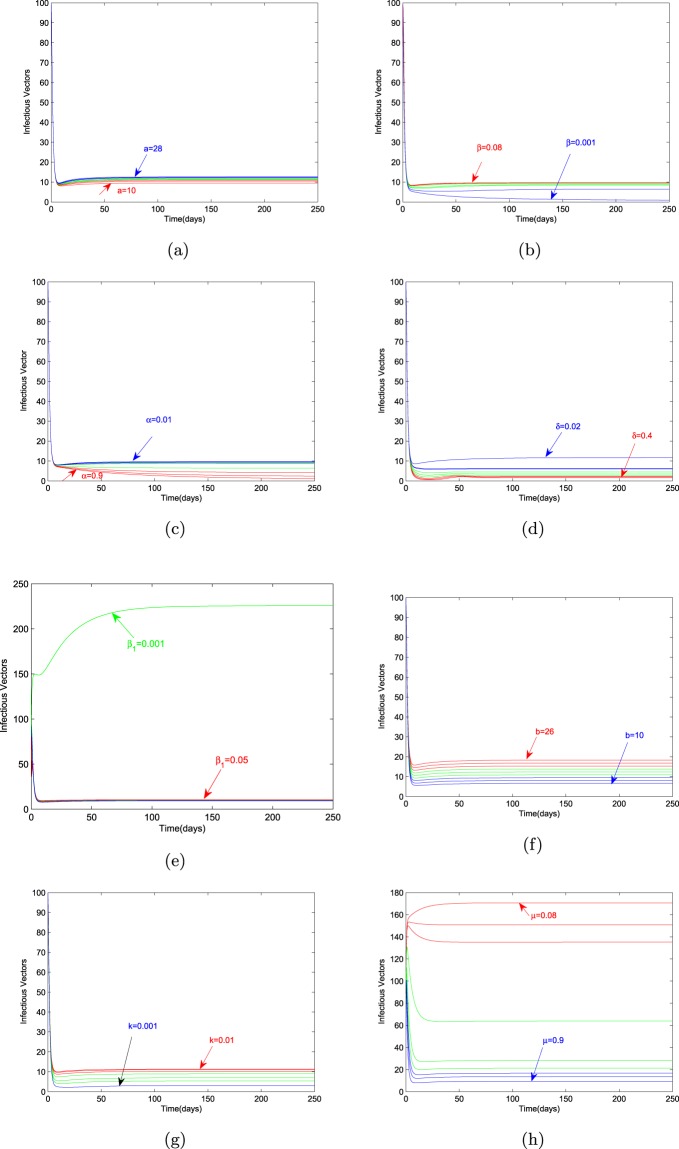
Variation in the endemic level of ${Y}^{\ast }$ with respect to model parameters.

It can be observed from the (Fig. 4) that the endemic level of infectious bark beetles is greatly influenced by the parameters ${\beta }_{1}$ and $\mu$. But the calculation of percentage difference of the values of ${Y}^{* }$ corresponding to percentage difference of the parametric values shows that the most sensitive parameter is again $\beta$. The value of this difference is $8890$. Percent increment or reduction in the value of ${Y}^{* }$ corresponding to all parameters is given in Table 2.

**Table 2:** Sensitivity of ${Y}^{* }$ for all model parameters:.

Parameters	Initial	Final	Difference	Percentage	Initial	Final	Difference	Percentage	${{\bf{C}}}_{{\bf{5}}}{\boldsymbol{/}}{\bf{91.11111111}}$	${{\bf{C}}}_{{\bf{9}}}\,{\boldsymbol{\ast }}\,{{\bf{C}}}_{{\bf{10}}}$
		Value		Difference	Value of ${Y}^{* }$	Value of ${Y}^{* }$		Difference		
$a$	10	28	18	180	10	13	3	30	1.97561	59.26829
$\beta$	0.001	0.08	0.079	7900	1	10	9	900	86.7073	78036.59
$\alpha$	0.9	0.01	-0.89	-98.88888889	1	9.5	8.5	850	-1.085366	-922.561
$\delta$	0.4	0.02	-0.38	-95	2	11	9	450	-1.042683	-469.207
${\beta }_{1}$	0.05	0.001	-0.049	-98	15	17	2	13.33333333	-1.075609756	-14.34146341
$b$	10	26	16	160	7	19	12	171.4285714	1.756097561	301.0452962
$k$	0.001	0.01	0.009	900	3	12	9	300	9.87804878	2963.414634
$\mu$	0.9	0.08	-0.82	-91.11111111	10	170	160	1600	-1	-1600

### Sensitivity indices

The sensitivity index helps to measure the relative change in the state variable when a parameter changes. The normalized forward sensitivity of a variable to the parameter is defined as the ratio of relative change in the variable to the relative change in the parameter^22^. When a variable is a differentiable function of the parameter, the sensitivity index may be defined in terms of partial derivatives.

#### Definition 1.

The normalized forward sensitivity index of a variable $x$, that depends differentiability on a parameter $p$, is defined as

$${\Gamma }_{p}^{x}=\frac{\partial x}{\partial p}\times \frac{p}{x}.$$

#### Sensitivity indices of ${R}_{\circ }$

The analytical expressions for the sensitivity indices of ${R}_{\circ }$ can be obtained by using the above definition. For example, the sensitivity index of ${R}_{\circ }$ with respect to $a$ is

$$\begin{array}{lll}\frac{\partial {R}_{\circ }}{\partial a} & = & 0.5\left(\sqrt{\frac{{a}^{2}{\gamma }^{2}}{{\mu }^{4}}+\frac{4abk\beta }{\alpha \delta {\mu }^{2}}}+\frac{a\gamma }{{\mu }^{2}}\right)\\ {\Gamma }_{a}^{{R}_{\circ }} & = & \frac{\partial {R}_{\circ }}{\partial a}\times \frac{a}{{R}_{\circ }}\\  & = & \frac{\left(a\alpha {\gamma }^{2}\delta +{\mu }^{2}\left(2bk\beta +\alpha \gamma \delta \sqrt{\frac{a(a\alpha {\gamma }^{2}\delta +4bk\beta {\mu }^{2})}{\alpha \delta {\mu }^{4}}}\right)\right)}{a\alpha {\gamma }^{2}\delta +{\mu }^{2}\left(4bk\beta +\alpha \gamma \delta \sqrt{\frac{a(a\alpha {\gamma }^{2}\delta +4bk\beta {\mu }^{2})}{\alpha \delta {\mu }^{4}}}\right)}.\end{array}$$

The above sensitivity index has no obvious mathematical structure so that we could decide whether ${R}_{\circ }$ is an increasing function of $a$ or decreasing function. However, when we use the numerical values of the parameters given in Table 2, we see that the sensitivity index of ${R}_{\circ }$ of the parameter $a$ is $0.51$. It means that when we increase the value of $a$ by $10 \%$, the value of ${R}_{\circ }$ increases by almost $5 \%$. Looking at the sensitivity indices of ${R}_{\circ }$ with respect to all the model parameters given in Table 3, we observe that the most sensitive parameter is $\mu$(the mortality rate of bark beetles), which has the highest value  $-1.012$. Negative sign shows that ${R}_{\circ }$ is a decreasing function of $\mu$. An increment in the value of $\mu$ by $10 \%$, the reduction in ${R}_{\circ }$ occurs by $10 \%$.

**Table 3:** Description of the model parameters and nominal values.

Parameters	Explanation	Value
$a$	Recruitment rate of *Monochamus alternatus*	10
$b$	Increase rate of Pines	14
$\beta$	Transmission rate of nematode through the maturation feeding of infected bark beetles	0.03
$\alpha$	Exploitation rate of Susceptible pines	0.03
$\delta$	Exploitation rate of infected pines	0.04
$k$	Trnasmission rate of *Bursaphelenchus xylophilus* through the contact of infectious pines and susceptible bark beetles	0.0058
${\beta }_{1}$	Trnasmission rate of nematode as a result of mating	0.01
$\mu$	Mortality rate of bark beetles	0.9

#### Sensitivity indices of ${I}^{* }$ and ${Y}^{* }$

The endemic level of infectious pines given in (3) is represented in the form of endemic level of infectious bark beetles ${Y}^{* }$ and it can be obtained by the quadratic equation (4). Therefore, we do not have an explicit expression for the endemic equilibrium. However, by using the values of parameters given in table 3, we can calculate the sensitivity indices of ${I}^{* }$ and ${Y}^{* }$.

The calculated values of sensitivity indices of ${I}^{* }$ given in Table 4, express that the most sensitive parameter for the endemic level of infectious pines is the parameter $\mu$. It is observed that the increment in the value of $\mu$ by $10 \%$, ${I}^{* }$ would decrease almost $16 \%$. The endemic level of infectious hosts is also an increasing function of $\beta$. By decreasing the value of $\beta$ by $10 \%$, ${I}^{* }$ would decrease almost $14 \%$.

**Table 4:** Values of sensitivity indices of ${R}_{\circ },{I}^{* }$ and ${Y}^{* }$.

Parameters	Sensitivity index of ${R}_{\circ }$	Sensitivity index of ${I}^{* }$	Sensitivity index of ${Y}^{* }$
$a$	0.51	0.0056	0.0000088
$b$	0.49	$-$0.31	1.0
$\beta$	0.49	1.45	$-$0.00070
$\alpha$	$-$0.49	$-$0.0018	$-$0.0016
$\delta$	$-$0.49	$-$1.14	$-$1.002
$k$	0.49	$-$0.31	$-$0.00049
${\beta }_{1}$	0.012	0.45	0.00071
$\mu$	$-$1.012	$-$1.60	$-$0.0025

Sensitivity indices of the endemic level of infectious bark beetles ${Y}^{* }$, given in Table 4, show that the parameters $b$ and $\delta$ play a vital role in the enhancement or reduction in the values of ${Y}^{* }$. ${Y}^{* }$ is an increasing function of $b$ and a decreasing function of $\delta$. Increment in the value of $\delta$ by $10 \%$, reduction in the value of ${Y}^{* }$ occurs by almost $10 \%$. Contrary to this, by decreasing the value of $b$  $10 \%$, ${Y}^{* }$ decreases by almost $10 \%$. To decrease the endemic level of infectious bark beetles, we cannot decrease the input rate of pines. However, we should focus on the felling rate of infectious pines in order to achieve the decreased endemic level of infectious bark beetles.

## Optimal Control

Sensitivity analysis urges to modify the model (1) to include some control measures for the specific counteractive action. The model including three controls ${u}_{1},{u}_{2}$ and ${u}_{3}$ is given as:

5 $$\begin{array}{lll}\frac{dS}{dt} & = & b-\beta (1-{u}_{1})YS-\alpha S,\\ \frac{dI}{dt} & = & \beta (1-{u}_{1})YS-\delta I-{r}_{0}{u}_{2}I,\\ \frac{dX}{dt} & = & a(1-{u}_{3})-k(1-{u}_{1})IX-{\beta }_{1}XY-\mu X-{r}_{1}{u}_{3}X,\\ \frac{dY}{dt} & = & k(1-{u}_{1})IX+{\beta }_{1}XY-\mu Y-{r}_{1}{u}_{3}Y,\,\,\,S\ge 0,\,\,I\ge 0,\,\,X\ge 0,\,\,Y\ge 0.\end{array}$$

The control ${u}_{1}(t)$ represents the utilization of nematocide infused into the stem of healthy trees, ${u}_{2}(t)$ shows the increase in the slashing rate of infected pine trees with the goal that bark beetle could not be able to oviposit on them and use of adulticide applied for vector control, for example, aerial spraying of pesticide, is expressed by the control function ${u}_{3}(t)$. To explore the ideal level of efforts to rein the ailment, we construct the objective functional $J$. Its purpose is to reduce the infectious trees and the cost of applied k8controls. One has

$$J({u}_{1},{u}_{2},{u}_{3})={\int }_{0}^{T}\left({A}_{1}I+{A}_{2}Y+\frac{{B}_{1}}{2}{u}_{1}^{2}+\frac{{B}_{2}}{2}{u}_{2}^{2}+\frac{{B}_{3}}{2}{u}_{3}^{2}\right)dt,$$

where ${A}_{1},{A}_{2}$ represent the positive weights. We will likely limit the number of infected trees figure while cutting down the expenditure of controls ${u}_{1}(t),{u}_{2}(t)$ and ${u}_{3}(t)$ with the aid of the above-mentioned objective functional. We shall find an optimal control ${u}_{1}^{* }$, ${u}_{2}^{* }$ and ${u}_{3}^{* }$ such that

$$J({u}_{1}^{* },{u}_{2}^{* },{u}_{3}^{* })=min\{J({u}_{1},{u}_{2},{u}_{3}),({u}_{1},{u}_{2},{u}_{3})\in U\},$$

where $U=\{({u}_{1},{u}_{2},{u}_{3})| {u}_{i}(t)\,is\,Lebesgue\,measurable\,on\,[0,1],0\ \le \ {u}_{i}(t)\ \le \ 1,i=1,2,3\},$ is the control set. The solution of this optimal control (OC) problem and derivation of necessary conditions are obtained by using the Pontryagin’s Maximum Principle^23^.

## Optimal Control Existence

Optimal Control existence can be demonstrated through a well known classical result: according to^24^, we must inspect that the following axioms are satisfied:

- (H1) Controls set and set of state variables are nonempty.
- (H2) The admissible control set $U$ is convex and closed.
- (H3) R.H.S of the state system is bounded by a linear function of the state variables and controls.
- (H4) The objective functional $J$ has convex integrand on $U$ and is bounded below by ${c}_{1}{({\sum }_{i=1}^{3}| {u}_{i}{| }^{2})}^{\frac{\beta }{2}}-{c}_{2}$, where ${c}_{1},{c}_{2} > 0$ and $\beta  > 1$.
- (H5) The existence of solutions for the system (5) is established by using the result given by Lukes (^25^, Th 9.2.1, p 182). In this way, we confirm the above hypothesis. $({H}_{1})$ is accomplished because the coefficients are bounded. The boundedness of solutions shows that the set of controls fulfills $({H}_{2})$. Since the system of equations is bilinear in ${u}_{1}$, ${u}_{2}$ and solutions are bounded. Hence, R.H.S of (5) meets the criteria ${H}_{3}$. The last condition is also satisfied because the integrand of the objective function is convex.

$${M}_{1}I+{M}_{2}Y+\frac{1}{2}{D}_{1}{u}_{1}^{2}+\frac{1}{2}{D}_{2}{u}_{2}^{2}+\frac{1}{2}{D}_{3}{u}_{3}^{2}\ge {c}_{1}{\left(\mathop{\sum }\limits_{i=1}^{3}| {u}_{i}{| }^{2}\right)}^{\frac{\gamma }{2}}-{c}_{2},$$

where ${M}_{1},{M}_{2},{D}_{1},{D}_{2},{D}_{3},{c}_{1},{c}_{2} > 0$ and $\gamma  > 1$. Thus we have the following theorem:

### Theorem 3.

*For the objective functional* $J({u}_{1},{u}_{2})={\int }_{0}^{T}{A}_{1}I+{A}_{2}Y+\frac{1}{2}({B}_{1}{u}_{1}^{2}+{B}_{2}{u}_{2}^{2}+{B}_{3}{u}_{3}^{2})dt$* where* $U=\{({u}_{1},$ ${u}_{2},{u}_{3})| 0\le \ {u}_{1},{u}_{2},{u}_{3}\ \le \ 1,t\in [0,T]$ *subject Eq*.(5) *with initial conditions, there exists an optimal control* $u=({u}_{1}^{* },{u}_{2}^{* },{u}_{3}^{* })$ so that $J({u}_{1}^{* },{u}_{2}^{* },{u}_{3}^{* })=min\{J({u}_{1},{u}_{2},{u}_{3}),({u}_{1},{u}_{2},{u}_{3})\in U\}$.

The optimal solution can be attained by finding the Lagrangian as well as Hamiltonian for the system (5). Its Lagrangian is

$${\mathbb{L}}(I,Y,{u}_{1},{u}_{2},{u}_{3})={A}_{1}I+{A}_{2}Y+\frac{1}{2}{B}_{1}{u}_{1}^{2}+\frac{1}{2}{B}_{2}{u}_{2}^{2}+\frac{1}{2}{B}_{3}{u}_{3}^{2}\ge {c}_{1}(| {u}_{1}{| }^{2}+| {u}_{2}{| }^{2}).$$

We have to set up the minimal value of the Lagrangian. For this purpose, we construct the Hamiltonian $H$ for OC problem as follows:

Let us take $${\mathcal{X}}\ =\ $$($S,I,X,Y$), $$\lambda \ =\ $$(${\lambda }_{1},{\lambda }_{2},{\lambda }_{3},{\lambda }_{4}$) and $$U\ =\ $$(${u}_{1},{u}_{2},{u}_{3}$), then we have

$$\begin{array}{lll}H({\mathcal{X}},U,\lambda ) & = & {A}_{1}I+{A}_{2}Y+\frac{1}{2}\mathop{\sum }\limits_{i=1}^{3}\frac{{B}_{i}}{2}{u}_{i}^{2}+{\lambda }_{1}(b-\beta (1-{u}_{1})YS-\alpha S)\\  &  & +{\lambda }_{2}(\beta (1-{u}_{1})YS-\delta I-{r}_{0}{u}_{2}I)\\  &  & +{\lambda }_{3}(a(1-{u}_{3})-k(1-{u}_{1})IX-{\beta }_{1}XY-\mu X-{r}_{1}{u}_{3})\\  &  & +{\lambda }_{4}(k(1-{u}_{1})IX+{\beta }_{1}XY-\mu Y-{r}_{1}{u}_{3}).\end{array}$$

### The optimality system

We apply the Pontryagin’s Maximum Principle^23^ for finding the essential conditions for this optimal control. It is done as follows:

*For the optimal solution*$({u}_{1}^{* },{u}_{2}^{* },{u}_{3}^{* })$*of OC problem* (5), *a non-zero vector function*$\lambda (t)$ exists that satisfies the subsequent conditions. The state equation, optimality condition and adjoint equation are given as respectively

$$\frac{dx}{dt}=\frac{\partial }{\partial \lambda }(H(t,{u}_{1}^{* },{u}_{2}^{* },{u}_{3}^{* },\lambda (t))),$$

$$0=\frac{\partial }{\partial u}(H(t,{u}_{1}^{* },{u}_{2}^{* },{u}_{3}^{* },\lambda (t))),$$

$$\frac{d\lambda }{dt}=-\frac{\partial }{\partial x}(H(t,{u}_{1}^{* },{u}_{2}^{* },{u}_{3}^{* },\lambda (t))).$$

The essential conditions applied to the Hamiltonian $H$ give the following result:

#### Theorem 4.

*For optimal controls* ${u}_{1}^{* },{u}_{2}^{* },{u}_{3}^{* }$and solutions ${S}^{* },{I}^{* },{X}^{* },{Y}^{* }$ of *the system* (5), *variables* ${\lambda }_{1},\ldots ,{\lambda }_{4}$* occurs having*

6 $$\begin{array}{lll}\frac{d{\lambda }_{1}}{dt} & = & \left({\lambda }_{1}-{\lambda }_{2}\right)\beta (1-{u}_{1})Y+{\lambda }_{1}\alpha ,\\ \frac{d{\lambda }_{2}}{dt} & = & -{A}_{1}+{\lambda }_{2}\left(\delta +{r}_{0}{u}_{2}\right)+\left({\lambda }_{3}-{\lambda }_{4}\right)k(1-{u}_{1})X,\\ \frac{d{\lambda }_{3}}{dt} & = & \left({\lambda }_{3}-{\lambda }_{4}\right)k(1-{u}_{1})I+\left({\lambda }_{3}-{\lambda }_{4}\right){\beta }_{1}Y+{\lambda }_{3}\mu +{\lambda }_{3}{r}_{1}{u}_{3},\\ \frac{d{\lambda }_{4}}{dt} & = & -{A}_{2}+\left({\lambda }_{3}-{\lambda }_{4}\right){\beta }_{1}X+{\lambda }_{4}\mu +{\lambda }_{4}{r}_{1}{u}_{3},\end{array}$$

with transversality conditions ${\lambda }_{1}(T)={\lambda }_{2}(T)={\lambda }_{3}(T)={\lambda }_{4}(T)=0$. Additionally, ${u}_{1}^{* },{u}_{2}^{* },{u}_{3}^{* }$ are expressed as

7 $$\begin{array}{lll}{u}_{1}^{* } & = & \max \{\min \{1,\frac{1}{{B}_{1}}\left(\left({\lambda }_{4}-{\lambda }_{3}\right)kIX+\left({\lambda }_{2}-{\lambda }_{1}\right)\beta YS\right)\},0\},\\ {u}_{2}^{* } & = & \max \{\min \{1,\frac{1}{{B}_{2}}{\lambda }_{2}{r}_{0}I\},0\},\\ {u}_{3}^{* } & = & \max \{\min \{1,\frac{1}{{B}_{3}}\left({\lambda }_{3}a+{\lambda }_{3}{r}_{1}X+{\lambda }_{4}{r}_{1}Y\right)\},0\}.\end{array}$$

*Proof*. The adjoint equations and the conditions of transversality are obtained by the Hamilton $H$. Set $S={S}^{* },I={I}^{* },X={X}^{* },Y={Y}^{* }$ and differentiate the Hamiltonian $H$ with respect to $S,I,{X}^{{\prime} }2,Y$. Thus we have

$$\begin{array}{lll}\frac{d{\lambda }_{1}}{dt} & = & \left({\lambda }_{1}-{\lambda }_{2}\right)\beta (1-{u}_{1})Y+{\lambda }_{1}\alpha ,\\ \frac{d{\lambda }_{2}}{dt} & = & -{A}_{1}+{\lambda }_{2}\left(\delta +{r}_{0}{u}_{2}\right)+\left({\lambda }_{3}-{\lambda }_{4}\right)k(1-{u}_{1})X,\\ \frac{d{\lambda }_{3}}{dt} & = & \left({\lambda }_{3}-{\lambda }_{4}\right)k(1-{u}_{1})I+\left({\lambda }_{3}-{\lambda }_{4}\right){\beta }_{1}Y+{\lambda }_{3}\mu +{\lambda }_{3}{r}_{1}{u}_{3},\\ \frac{d{\lambda }_{4}}{dt} & = & -{A}_{2}+\left({\lambda }_{3}-{\lambda }_{4}\right){\beta }_{1}X+\left({\lambda }_{1}-{\lambda }_{2}\right)\beta (1-{u}_{1})S+{\lambda }_{4}\mu +{\lambda }_{4}{r}_{1}{u}_{3},\end{array}\,$$

with transversality conditions ${\lambda }_{1}(T)={\lambda }_{2}(T)={\lambda }_{3}(T)={\lambda }_{4}(T)=0$. The conditions of optimality and the property of control set $U$ give the result

□ $$\begin{array}{lll}{u}_{1}^{* } & = & \max \{\min \{1,\frac{1}{{B}_{1}}\left(\left({\lambda }_{4}-{\lambda }_{3}\right)kIX+\left({\lambda }_{2}-{\lambda }_{1}\right)\beta YS\right)\},0\},\\ {u}_{2}^{* } & = & \max \{\min \{1,\frac{1}{{B}_{2}}{\lambda }_{2}{r}_{0}I\},0\},\\ {u}_{3}^{* } & = & \max \{\min \{1,\frac{1}{{B}_{3}}\left({\lambda }_{3}a+{\lambda }_{3}{r}_{1}X+{\lambda }_{4}{r}_{1}Y\right)\},0\}.\end{array}$$

### Experimental results of optimal control

We now solve the OC problem numerically and show some results. Since the initial values of the state variables $S,I,X$ and $Y$ are given, therefore, forward RK-4 method is applied to solve the system of equations (5). The backward RK-4 method is applied to solve the adjoint system (6) because final values are given. Regarding the control variables, an initial guess is given. Time-varying control variables have been found by putting the values of the state and adjoint variables in the system (7). We consider that the optimal campaign continues for 120 days. We present the use of different controls.

#### Applications of two controls

**Figure 5:**
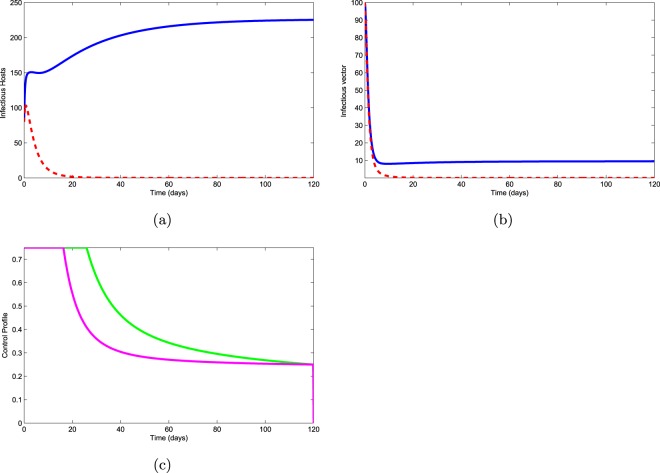
Use of controls ${u}_{1}$ and ${u}_{2}$.

Application of nematocide $({u}_{1}\ \ne \ 0)$ and slashing of infected pines (${u}_{2}\ \ne \ 0$): In this strategy, only two controls, nematocide and slashing of infected pine trees, are used to optimize the objective functional $J$, whereas third control (aerial spraying) is set to zero. Figure 5a shows that a considerable decrease occurs in the population of infectious pines and according to Fig. 5b small decrease appears in the population of vectors. The control profile is given in Fig. 5c. From this control profile, we observe that nematocide has to apply continuously for 26 days with great animation and then we can decrease this effort. From Fig. 5c, it can also be seen that we should make a maximum effort to slash the infected pines for 18 days and then slow down this control up to 120 days.

**Figure 6:**
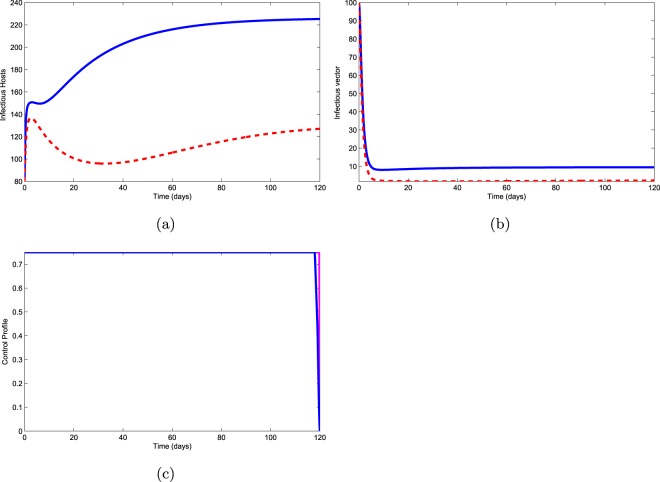
Use of controls ${u}_{1}$ and ${u}_{3}$.

Use of nematocide (${u}_{1}\ne 0$) and aerial spraying (${u}_{3}\ne 0$): This control policy is based on the use of two controls, nematocide and aerial spraying. Figure 6a shows that infectious pines slightly increases first, and then began to decrease for 40 days. After this period the number of infectious pines increases up to 120 days. Figure 6b shows that infectious vectors decrease to zero after 7 days. Control profile is given in Fig. 6c shows that we have to use nematocide and aerial spraying for 120 days continuously. However, Fig. 6a,b show the non-suitable strategy.

**Figure 7:**
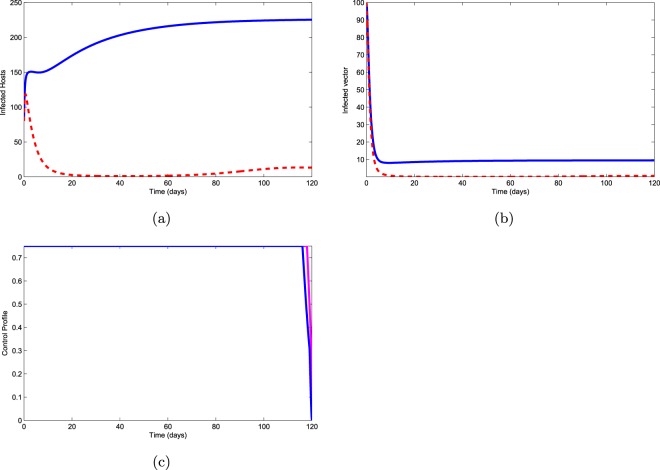
Use of controls ${u}_{2}$ and ${u}_{3}$.

Slashing of infected pines (${u}_{2}\ne 0$) and aerial spraying (${u}_{3}\ne 0$): In this policy, slashing of infected pines and aerial spraying are used to optimize the objective functional $J$. Fig. 7a shows that the application of this strategy minimizes infectious pines first but after 80 days they again start to increase. Infectious vectors decrease to zero as shown in Fig. 7b. The control profile showing in Fig. 7c informs that we have to apply this control strategy for 118 days with great animation. However, Fig. 7a shows that this is not an effective control strategy because the infectious pines increase ultimately.

#### Application of three controls

**Figure 8:**
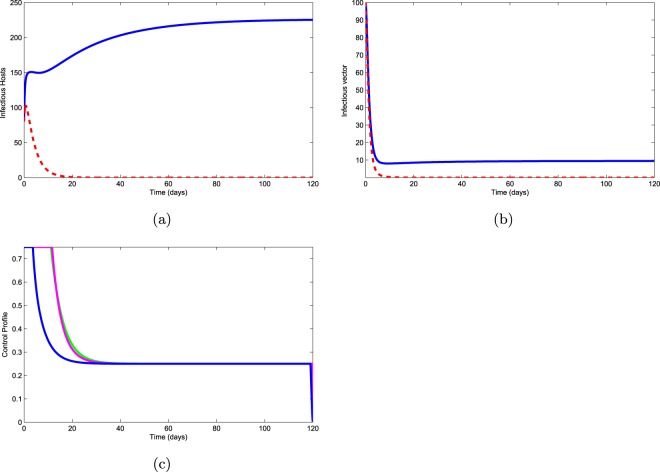
Use of three controls.

In this strategy, all control measures are used to optimize the objective functional $J$. Figure 8a shows that infectious pines decrease to zero after 18 days. The number of infected bark beetles also decreases to zero after 7 days as shown in Fig. 8b. This is the best control strategy because from Fig. 8c, we see that aerial spraying should apply for 5 days and nematocide and slashing of infected pines should be applied for 15 days with full effort. Since the period of application of these controls with great animation is short, therefore, the cost of these applied controls is reasonable.

## Conclusions

The deterministic model of pine wilt disease has been rigorously analyzed. In this article, the global behavior of equilibria has been discussed on the basis of basic reproduction. Global stability of disease-free equilibrium has been proved by constructing a suitable Lyapunov function whereas the global behavior of endemic equilibrium has been investigated by using the graph-theoretic approach. Two features have been discussed in the sensitivity analysis. Firstly, it has been observed that how the endemic level of infectious hosts and vectors is effected by varying the values of parameters. Secondly, the corresponding change in the state variable has been measured when a parameter changes. When we calculate the percentage difference of the values of ${I}^{* }$ and ${Y}^{* }$ corresponding to the percentage difference of the parameter values, it is observed that the most sensitive parameter is $\beta$ for ${I}^{* }$ as well as for ${Y}^{* }$. The calculation of sensitivity indices of ${R}_{0}$ has shown that the most sensitive parameter for ${R}_{0}$ is the mortality rate of bark beetles. The extreme values of the sensitivity indices of ${I}^{* }$ and ${Y}^{* }$ are $-1.60$ and $-1.002$ corresponding to the parameter values $\mu$ and $\delta$, respectively. These sensitivity indices show that by increasing the mortality rate of bark beetles by $10 \%$, ${I}^{* }$ would decrease almost $16 \%$. Similarly, index $-1.002$ shows that the increment of the slashing of infected pines by $10 \%$ results reduction in the endemic level of ${Y}^{* }$ by $10 \%$. The sensitivity analysis has shown us to design optimal control problem having three controls, namely, the use of nematocide implanted into the trunk of healthy trees, the increase in felling rate of affected pine trees so that bark beetle could not succeed to oviposit on them and the level of adulticide proposed for vector control such as aerial spraying of pesticide.

## References

[1] Zhao, B. G., Futai, K., Jack, R., Sutherland, J. R. & Takeuchi, Y. *Pine Wilt Disease*. (Springer, 2008).

[2] Mamiya Y (1983). Pathology of the pine wilt disease caused by Bursaphelenchus xylophilus. Annu. Rev. Phytopathol..

[3] Kiyohara T, Tokushige Y (1971). Inoculation experiments of a nematode, Bursaphelenchus sp., onto pine trees. J. Jap. Forest Soc..

[4] Mamiya Y, Kiyohara T (1972). Description of Bursaphelenchus lignicolus n. sp. (Nematoda: Aphelenchoididae) from pine wood and histopathology of nematode-infested trees. Nematologica.

[5] Nickle WAR, Golden AM, Mamiya Y, Wergin WP (1981). On the taxonomy and morphology of the pine wood nematode, Bursaphelenchus xylophilus (Steiner and Buhrer 1934) Nickle 1970. J. Nematol..

[6] Mamiya Y, Enda N (1972). Transmission of Bursaphelenchus lignicolus (Nematoda: Aphelenchoididae) by Monochamus alternatus (Coleoptera: Cerambycidae). Nematologica.

[7] Wingfield MJ, Blanchette RB (1983). The pine-wood nematode, Bursaphelenchus xylophilus, in Minnesota and Wisconsin: insect associates and transmission studies. Can. J. Forest. Res..

[8] Arakawa Y, Togashi K (2002). Newly discovered transmission pathway of Bursaphelenchus xylophilus from males of the beetle Monochamus alternatus to Pinus densiflora trees via oviposition wounds. J. Nematol..

[9] Yoshimura A (1999). Modeling the spread of pine wilt disease caused by nematodes with pine sawyers as vector. Ecology.

[10] Takasu F (2000). Modeling the expansion of an introduced tree disease. Biol. Invasions.

[11] Takasu F (2009). Individual-basedmodeling of the spread of pine wilt disease: vector beetle dispersal and the Allee effect. Popul. Ecol..

[12] Ozair, M. Analysis of pine wilt disease model with nonlinear incidence and horizontal transmission. *J. Appl. Math*.** 2014**, (2014).

[13] Ozair M, Shi X, Hussain T (2016). Control measures of pine wilt disease. Comput. Appl. Math..

[14] Awan AU, Ozair M, Din Q, Hussain T (2016). Stability analysis of pine wilt disease model by periodic use of insecticides. J. Biol. Dynam..

[15] Awan AU, Hussain T, Okosun KO, Ozair M (2018). Qualitative analysis and sensitivity based optimal control of pine wilt disease. Adv. Differ. Equ-NY..

[16] Khan MA (2017). Mathematical modeling and stability analysis of pine wilt disease with optimal control. Sci. Rep-UK..

[17] Shi, X. & Guohua, S. Analysis of the mathematical model for the spread of pine wilt disease. *J. App. Math*.**2013**, (2013).

[18] Togashi K, Arakawa Y (2003). Horizontal transmission of Bursaphelenchus xylophilus between sexes of Monochamus alternatus. J. Nematol..

[19] Driessche PVD, Watmough J (2002). Reproduction numbers and sub-threshold endemic equilibria for compartmental models of disease transmission. Math. Biosci..

[20] Shuai Z, Driessche PVD (2013). Global stability of infectious diseases models using lyapunov functions. SIAM J. App. Math..

[21] LaSalle, J. P. The stability of dynamical systems. Regional Conference Series in Applied Mathematics. SIAM, Philadelphia. (1976).

[22] Chitnis N, Hyman JM, Cushing JM (2008). Determining important parameters in the spread of malaria through the sensitivity analysis of a mathematical model. B. Math. Biol..

[23] Pontryagin, L. S., Boltyanskii, V. G., Gamkrelidze, R. V. & Mishchenko, E. F. The Mathematical Theory of Optimal Processes. (Gordon and Breach Science Publishers, 1986).

[24] Fleming, W. H. & Rishel, R. W. Deterministic and Stochastic Optimal Control. (Springer, 1975).

[25] Lukes, D. L. Differential Equations: Classical to Controlled. (Academic Press, 1982).

